# Vectorial principles of sensorimotor decoding

**DOI:** 10.3389/fnhum.2025.1612626

**Published:** 2025-07-07

**Authors:** Vassiliy Tsytsarev, Anna Volnova, Legier Rojas, Priscila Sanabria, Alla Ignashchenkova, Jescelica Ortiz-Rivera, Janaina Alves, Mikhail Inyushin

**Affiliations:** ^1^Department of Anatomy and Neurobiology, School of Medicine, University of Maryland, Baltimore, MD, United States; ^2^Institute of Translational Biomedicine, Saint Petersburg State University, Saint Petersburg, Russia; ^3^School of Medicine, Central University of the Caribbean, Bayamón, Puerto Rico; ^4^Nevsky Center of Scientific Collaboration, Saint Petersburg, Russia

**Keywords:** sensorimotor system, motor control, sensory systems, perception, sensory and motor coding

## Abstract

This review explores the vectorial principles underlying sensorimotor decoding across diverse biological systems. From the encoding of light wavelength in retinal cones to direction-specific motor cortex activity in primates, neural representations frequently rely on population vector coding–a scheme, in which neurons with directional or modality-specific preferences integrate their activity to encode stimuli or motor commands. Early studies on color vision and motor control introduced concepts of vector summation and neuronal tuning, evolving toward more precise models such as the von Mises distribution. Research in invertebrates, including leeches and snails, reveals that even simple nervous systems utilize population vector principles for reflexes and coordinated movements. Furthermore, analysis of joint limb motion suggests biomechanical optimization aligned with Fibonacci proportions, facilitating efficient neural and mechanical control. The review highlights that motor units and neurons often display multimodal or overlapping tuning fields, reinforcing the need for population-based decoding strategies. These findings suggest a unifying vectorial framework for sensory and motor coding, with implications for periprosthetic and brain-machine interface.

## 1 Introduction: population vectors as a common language of the nervous system

The concepts of multiple neuron participation in coding, neuronal arcs, and neuronal populations have evolved over time. Early electrophysiological studies focused on recording individual neurons and examining their responses to different stimuli, leading to a “classical” view of neuronal coding, where the modulation of firing rates influenced neuronal output. However, it eventually became clear that individual neurons are part of a larger network responsible for coding. Two key concepts emerged: (1) Neuronal Arc: Neurons are hierarchically interconnected with direct and feedback loops. These connections can involve sensory elements, interneurons, and effectors, forming open or closed loops known as arcs. (2) Multineuronal Arc (Neuronal Network): Multiple neurons work in parallel, with their integrative action creating a coordinated output. This concept was articulated by Levine (2007). Additionally, the trichromatic theory of color vision was developed by Young and von Helmholtz and was later advanced by Schrödinger suggesting that three sets of specialized neurons simultaneously code color perception, forming a vector space (Niall, 2017; Feynman et al., 1963). Interestingly, initial visual processing in the retina includes signal decorrelation, generating linearly independent color vectors that likely form part of an orthogonal basis for color perception (Iniushin and Stankevich, 2001).

The first neuronal net-type model for codifying and recognizing patterns was developed (McCulloch and Pitts, 1943). Additionally, the idea of neuronal populations processing information in groups was proposed through cell assembly theory (Attneave and Hebb, 1950). Over time, it became clear that sensory information could be represented using combinatorial systems, akin to vector coding, known as Parallel Distributed Processing (Churchland, 1995). This vector coding system enables precise analysis and recognition of sensory inputs. The brain processes neuronal activation patterns through synaptic connections in a way similar to vector transformations. Synapses modify input patterns (vectors) to produce output patterns (also vectors). Parallel Distributed Processing involves simultaneous computations across networks, much like parallel vector processing. Similar ideas related to vector coding have been developed worldwide (Sokolov and Vatkyavichus, 1988). Vector-based concepts in neurophysiology have become more advanced with the introduction of the neuronal population vector, which plays a key role in sensorimotor decoding (Georgopoulos et al., 1983; Mahan and Georgopoulos, 2013; Pais-Vieira et al., 2023). It was Georgopoulos et al. who demonstrated a clear neuronal implementation: the direction of a primate’s arm movement could be predicted by summing vectors aligned with the preferred directions of individual motor-cortex neurons. This concept involves three key elements: a behavioral measure represented in multidimensional space, a neuronal population, and an orderly variation in the neural activity of the neurons within that population corresponding to changes in the behavioral measure. The computation is a weighted vector sum of neural activities, providing an estimate of the behavioral outcome. This idea closely echoes Sherrington’s earlier concepts of neural coordination. This area of research is rapidly evolving, challenging traditional ideas, and undergoing significant transformation. In this context, we review the literature on sensorimotor vector decoding and examine the specific studies that display the foundation for these ideas.

Roadmap of the review: Section 1 introduces foundational vector concepts; Section 2 surveys exemplar sensory systems; Section 3 discusses motor implementations; Section 4 provides an overview of vector-motoneurons; Section 5 provides an integrative Discussion and future directions; and Section 5.1 summarizes Conclusions.

## 2 Coding of color frequency by the population of specialized sensory neurons (cones) in the vertebrate retina and the trichromatic theory of color vision

Newton’s seminal experiments with prisms, documented in his 1,704 work Opticks, demonstrated that sunlight could be refracted into a spectrum of colors when passed through a glass prism, and proved that white light itself is a composite of all visible hues (Newton, 2010). By isolating individual colors and recombining them using lenses and secondary prisms, he regenerated white light, conclusively showing that color arises from the separation and interaction of light’s constituent wavelengths, thus establishing the continuous nature of the color gradations and introducing wavelength scale. Refracting sunlight through prisms, he demonstrated that white light decomposes into a constant spectrum of hues, which he arbitrarily divided into seven colors (ROYGBIV) for symbolic alignment with musical scales but identified three primary colors–red, green, and indigo (blue) (RGB)–based on their capacity to regenerate white light when combined.

This insight marked the birth of additive color theory, where light wavelengths are superimposed to create new hues. He also introduced Newton’s circular color wheel (Circle of Colors), the first graphical representation of color relationships, positioning these primaries opposite their complementary colors (e.g., red opposite cyan), illustrating that pairs of complementary lights mix to produce white. To explain primary colors and additive color theory, Thomas Young in 1802 proposed a biological basis for trichromacy, that the eye contains three types of “particles” (later termed cones) sensitive to distinct portions of the spectrum. Drawing direct inspiration from Newton’s additive primaries, Young hypothesized that each receptor type responds preferentially to red, green, or violet (blue) light. This triadic model mirrored Newton’s observation that three spectral primaries suffice to simulate all perceived colors through additive mixing. After establishing Christiaan Huygens’ wave theory, it was already known that the visible spectrum spans wavelengths from approximately 400 (violet) to 700 nm (red), with each hue corresponding to a specific range, and Young suggested that color is the visible manifestation of light’s wavelength.

Hermann von Helmholtz expanded Young’s theoretical framework through rigorous psychophysical experiments in the 1,850’s–60’s. Using color-matching tasks, he demonstrated that observers could replicate any hue by adjusting the intensity of three monochromatic lights–red (long wavelength), green (medium), and blue-violet (short). He also accurately quantified spectral sensitivity, showing the non-linearity of trichromat space. Helmholtz recognized that perceptual color differences do not map linearly to physical wavelengths, prompting his exploration of Riemannian metrics–a mathematical tool for describing curved spaces. He introduced a line element to correlate perceptual just-noticeable differences (JNDs) with infinitesimal distances in a 3D color space (von Helmholtz and Southall, 1962).

Genius physicist Maxwell, practically at the same time (1,857), in a series of similar color-mixing psychophysical experiments, confirmed Newton’s color-additive theory and the near linearity of three principal colors. Moreover, speaking on a graphical method of exhibiting the relations of colors suggested that “the method which exhibits to the eye most clearly the results of this theory of the three elements of color is that which supposes each color to be represented by a point in space, whose distances from three co-ordinate planes are proportional to the three elements of color,” and “this requiring space of three dimensions.” Maxwell (1857) also decided, that Newton’s Circle of Colors and Mayer and Young’s Color Triangle and any method by which the operations are confined to a plane “has been adopted for convenience” only.

Another famous name in the development of the trichromatic theory was Erwin Schrödinger, best known for his foundational contributions to quantum mechanics. His foray into color theory during the 1920’s positioned him as an intellectual successor to Helmholtz. In his 1920 papers, entitled Grundlinien einer Theorie der Farbmetrik im Tagessehen (Foundations of a Theory of Color Metrics in Daylight Vision), Schrödinger revisited Helmholtz’s 1,891–92 attempts to model color space using Riemannian geometry. Helmholtz had proposed a non-Euclidean line element to quantify perceptual color differences, but his model faced mathematical inconsistencies. Schrödinger posited that adaptation to illuminants corresponds to linear automorphisms of color space, preserving the cone’s convex structure, and his refinements resolved issues, cementing Helmholtz’s intuitive leap into a rigorous framework (Niall, 2017; Provenzi, 2020). Another Nobel in Physics, Richard Feynman, popularized 3D-vector color space in Volume 1 of his famous Lectures on Physics, mentioning Schrödinger’s work, and providing an accessible, physics-oriented exposition of Schrödinger’s color-metric, fostering adoption in neuroscience (Feynman et al., 1963).

**FIGURE 1:**
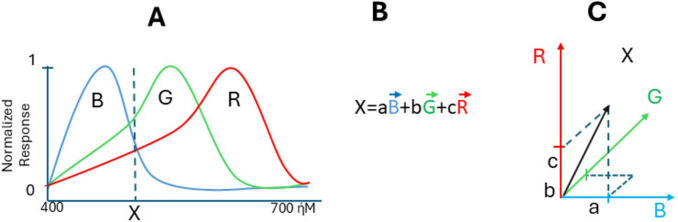
Color frequency coding in trichromat retina. **(A)** Normalized responses of cone cells to the stimulus of different wavelength, **(B)** formula of response of cones to the stimulus of X-wavelength, **(C)** visualization of vector X in three-dimensional space with vectors R, G, and B with components a, b, and c. The cone-sensitivity curves are taken from Fukurotani (1982), with permission.


Nowadays, the data on the structure and functioning of color channels in the eye retina are confirmed by modern physiology, with microspectrophotometry of all types of cones and characterization of opsins and their genes, including human opsins. Speaking about vertebrate animals, some are dichromatic (like male marmoset monkeys, which have only short-wavelength “S” cones and a single type of medium/long-wavelength “M/L” cones (Solomon and Rosa, 2014), while others, like turtles, have tetrachromatic vision with cones specifically sensitive to different wavelengths, including red, green, blue, and ultraviolet (UV) light, suggesting that there is a separate ultraviolet channel and a neural basis for tetrachromacy (Fuortes et al., 1973; Ventura et al., 2001). Many vertebrates have trichromatic eyes because the retina catches the light frequency by specialized sensory neurons (cones), with maximal sensitivity to long (R), middle (G), or short (B) wavelengths (their normalized response curves versus wavelength are shown in Figure 1A for carp fish retina (*Cyprinus carpio*). Suppose there is light with a wavelength X entering the eye (dash line on graph). In that case, it stimulates all three cones, each of them producing the response, with their specific intensity so that color X could be made by certain amounts of these three: say an amount a of blue color (aB), an amount b of color G (bG), and an amount c of color R (cR) makes X. We can write:

X=aB+bG+cR⁢(see⁢it⁢in⁢Figure 1B).

It may be interpreted as calculating a “population vector.” By conceptualizing color perception within a vector space framework, it becomes clear how different colors and their mixtures arise from the interaction and relative intensity of signals from R-, B-, and G-cones. If there is another color Y, it will produce the response in cones with intensities:


$$Y=a^{\prime }\,B+b^{\prime }\,G+c^{\prime }\,R$$

Then, the mixture of the two lights is obtained by taking the sum of the components of X and Y:


Z=X+Y=(a+a)′B+(b+b)′G+(c+c)′R

This is according to Newton’s additive primaries rule, and it is a standard vector summation formula. So, the trichromatic theory of color vision is fundamentally vector-related due to its reliance on three types of cone cells in the retina, each sensitive to different wavelengths of light. The relationship between these colors can be understood through the concept of vector space, and according to Maxwell, color X can be better visualized in three-dimensional space with vectors R, G, and B with components a, b, and c (Figure 1C). While the problem of non-linearity of RGB vector space persists, the brain resolves it through the reduction of statistical redundancy, without Riemannian geometry. One can see that responses R, G, and B on the cones level are correlated because activating B, for example, also activated G and R to some extent. Barlow and Földiák (1989) hypothesized that the role of early sensory neurons is to remove statistical redundancy in the sensory input, which can be achieved through the concept of coding efficiency (Simoncelli and Olshausen, 2001). Confirming that, it was shown that on the level of horizontal cells (next retinal layer), color functions became linearly independent and orthogonal, forming a perfect linearly independent color basis (Iniushin and Stankevich, 2001; Fukurotani, 1982). Such decorrelation and orthogonalization happen due to inhibitory feedback between retinal layers (Iniushin and Stankevich, 2001). It also happens in many other neural networks; their initial layers have correlated spike rates over time and/or correlated receptive fields. Decorrelation in biological neural networks refers to a tendency for neurons to compete and reduce redundancy in the network’s representation of sensory input due to inhibitory feedback (Tetzlaff et al., 2012). Now, decorrelation is an important stage of data processing in artificial networks.

One can see that the retina is using neuronal decoding methods such as the “population vector” to decode light frequency. The population vector represents the sum of a population of neurons’ “preferred” responses, weighted by their respective reaction (graded change in membrane potential or respective spike counts, etc.), allowing for the encoding of any stimulus position on the wavelength scale (Figure 1B). A similar coding strategy is most obvious in biological networks calculating the “preferred direction” of movements (Georgopoulos et al., 1983).

Modern researchers have expanded our understanding of how additional visual modalities are represented in the visual cortex, revealing that each neuron can be interpreted as encoding a vector (Gilbert and Wiesel, 1990; Vogels, 1990). In this updated framework, a neuron’s preferred orientation determines the direction of the vector, while its firing rate corresponds to the vector’s magnitude. The collective activity of the neuronal population represents the stimulus orientation through the vector sum of all individual neuronal responses–an approach known as the population vector coding scheme.

Later, similar vectorial coding was proposed for olfactory stimuli (odors) (Schild, 1988; Berglund and Olsson, 1993; Laing et al., 1994), and gestational stimuli (test) (Di Lorenzo, 1989; Schifferstein and Frijters, 1993) and other sensory modalities follow (Ince et al., 2013; Tanabe, 2013; Failor et al., 2025). Thus, tonotopy refers to one of the key properties of the auditory system (Tsytsarev and Tanaka, 2002). The essence of this phenomenon is the systematic arrangement of neurons in the auditory system by their functional preference. This means that neurons that respond to similar frequencies are located next to each other in different brain structures. Functional tonotopic maps are located throughout the auditory tract, starting in the cochlea and continuing into the brainstem, midbrain, thalamus, and auditory cortex. The sensitivity of neurons to sensory stimulation at each level of the auditory cortex is different, and each neuron has its own “best frequency”–the frequency at which the neuron responds to an auditory stimulus of minimal amplitude. As the frequency increases or decreases, the neuron’s sensitivity decreases. Things are a bit more complicated with other properties of sound, in particular, with the location of the sound source (Tsytsarev et al., 2009; Dalmas et al., 2024). The brain utilizes interaural time differences (ITD) and interaural level differences (ILD) to localize the sound source (Schmid et al., 2023). The medial superior olive (MSO) and lateral superior olive (LSO) in the brainstem play a crucial role in this process (Tollin, 2003). There is evidence for the presence of topographic representations of auditory space in the brain, but the proof of such functional maps is ambiguous. Certainly, the brain creates topographic maps of the location of sound sources in space, and individual neurons encode information about the sound source, but the structure of these maps is complex and not fully understood.

Evidence of topographic representations of auditory space in the brain dates back to the end of the last century, but it would be wrong to call these representations maps–their organization is more complex. Thus, it was shown that the external nucleus of the inferior colliculus (ICX) of the pigmented guinea pig contains a map of auditory space (Binns et al., 1992). Electrophysiological studies of neural clusters in the ICX to threshold and near-threshold stimuli have demonstrated acute spatial tuning.

The auditory system localizes the source of a sound based on the analysis of several parameters of sound signals (Tsytsarev et al., 2009). This analysis begins in the tonotopic pathway, then frequency-specific information is processed in the midbrain and forebrain. Higher-order neurons are tuned to specific locations in space (Cohen and Knudsen, 1999).

In the midbrain, space is represented as a kind of map, while in the forebrain, space is represented as clusters of similarly configured neurons (Cohen and Knudsen, 1999). The location of the sound source is represented in the brain in a rather complex way. It can be said that these representations are dynamic in nature, since the neurons that form them are capable of rapid reconfiguration and change in their functional properties.

These representations reach even greater complexity in animals capable of echolocation (Hagemann et al., 2010). Thus, topographic cortical representations of echo delay are present in the auditory cortex of some bats. Such cortical echo delay maps provide a calibrated neural representation of the spatial distance of an object (Hagemann et al., 2010).

## 3 Encoding the location of a touch stimulus by the population of mechanosensory neurons from the segmental ganglion of the leech and leech local bending reflex

**FIGURE 2:**
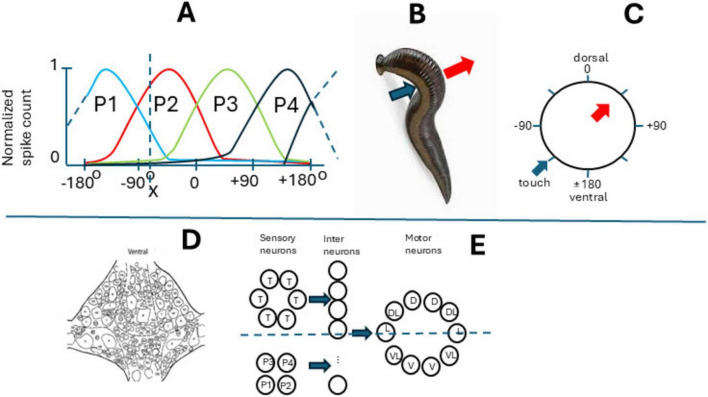
Leech and the local bending reflex. **(A)** Normalized responses of P-cells to the touch of the body wall, **(B)** the touch (blue arrow) and the bent (red arrow). **(C)** The circular body wall is shown in polar coordinates (grades) from 0 (dorsal) to 180 (ventral); arrows show the same as in **(B)**. **(D)** Cells in the segmental ganglion (ventral view), **(E)** cells in the segmental bent network (inhibitory neurons not shown). Motoneurons are marked as follows: V-ventral, VL-ventrolateral, D-dorsal, DL-dorsolateral, L-lateral. X-the point where the body wall is touched.

The medicinal leech (*Hirudo medicinalis*) body plan consists of 21 midbody segments with one ganglion per segment and a corresponding nerve cord. Leeches possess a compact, accessible nervous system with individually identifiable neurons and stereotyped behaviors, enabling interesting findings at a cellular level. Its population-vector touch response offers a uniquely transparent model for linking single-neuron activity to whole-body sensory coding, studied by Dr. William Kristan and his coauthors. A moderate mechanical stimulus applied to the leech’s body surface induces a localized withdrawal response at the stimulation site, a body bend directed away from the touch site (Lockery and Kristan, 1990; Lewis and Kristan, 1998b). This response is mediated by the contraction of longitudinal muscles at the stimulated location while the muscles on the opposite side relax, producing a U-shaped bend (see Figure 2B). Consequently, a stimulus on the dorsal side elicits a dorsal bend, a ventral stimulus results in a ventral bend, and a lateral stimulus leads to a lateral bend (see Figure 2C, showing body perimeter with arrows showing the stimulus location and bending direction). The local bending reflex can be triggered within a single segmental ganglion, indicating that each of the 21 segmental ganglia in the leech possesses the necessary neurons to generate this behavior (see Figure 2D showing ventral view of segmental ganglion): sensory, interneurons, and motor neurons representing three neuronal levels (Figure 2E). Many of these neurons are accessible with microelectrodes from the ventral part of the ganglion in four-segment semi-intact preparation (Lewis and Kristan, 1998b). Each ganglion contains sensory neurons that respond to touch (T cells) and pressure (P cells), with overlapping receptive fields (Nicholls and Baylor, 1968). Specifically, there are three pairs of T cells, each selectively responsive to touch on different body surfaces, encoding the velocity of a stimulus, and two nociceptive cells. Additionally, two pairs of P cells detect pressure, with one pair responding mainly to dorsal (P3, P4) and the other to ventral (P1, P2) stimulation, their responses distributed evenly around the body perimeter. Seven distinct classes of motor neurons regulate the longitudinal muscles involved in the reflex (Figure 2A). These include 4 excitatory and two inhibitory neurons for the ventral and similar ones for dorsal lateral muscles, and a pair of L-cells (excitatory), which have a combined dorsal and ventral field. Each motor neuron type innervates only one side of the body (left or right). The sensory neurons activate a layer of interneurons that activate a layer of motor neurons (Lewis and Kristan, 1998b; Lewis and Kristan, 1998a).

Because of the small size of this network, authors monitored and manipulated the complete set of sensory P-inputs to the network. The authors found that these neurons exhibit cosine-shaped tuning curves for stimulus location, with their peaks, or preferred stimulus locations, evenly distributed around the body perimeter (Figure 2A). Using a previously established neural decoding method, they estimated that stimulus location could be encoded in the spike counts of the four P neurons with a root-mean-squared (r.m.s.) error of just 3% (expressed as a percentage of 360°). In contrast, the local bending behavior was directed within 8% (r.m.s.) of the actual stimulus location. The higher accuracy of the P neuron representation compared to the behavioral response suggests that the local bend network could utilize the spike count-based population code of P neurons.

Neural networks with evenly distributed tuning curves are particularly well-suited for processing directional information and may be analyzed using neuronal decoding methods such as the “population vector.” The population vector represents the sum of a population of neurons’ preferred directions, weighted by their respective spike counts, allowing for the encoding of any stimulus location. The population vector serves as an optimal decoding method in the case of P neurons, which exhibit near cosine tuning and whose preferred stimulus locations form a two-dimensional Cartesian coordinate system. For example, if we touch the body wall in position X (see Figure 2A), we stimulate mainly neurons P1 and P2, but to some small extent also P3 and P4. We can write:

X = aP1+bP2+cP3+dP4, where c and d are near zero, and X is the specific response of the “population” in point X. And yes! It is the same vectorial summation formula

To eliminate the possibility of other coding methods, like winner-takes-all or simple averaging, authors simultaneously activated two P -cells using intracellular stimulation and found that the response corresponded to population vector summation. Also, they analyzed the connection of P-cells to 17 different interneurons. Authors found out that the synaptic strength from P neurons to each of the 17 identified local bending interneurons was proportional to the cosine of the difference in their preferred stimulus locations, confirming the pattern of connections that results in the accurate transfer of information encoded in a neural population vector (Lewis and Kristan, 1998a). Thus, the leech’s interneurons process the information from mechanosensory cells by calculating a “population vector.” This vector is formed by summing the preferred directions of each mechanosensory neuron, weighted by their activity levels. This population vector accurately encodes the direction to move away from the stimulus, providing a reliable mechanism for spatial orientation.

Similar tactile representations in mammals may use multiple populations of neurons in different regions of the brain (Nicolelis et al., 1998). A vector is a mathematical entity characterized by both magnitude (size) and direction. The population vector concept for motor-related neurons (neurons that determine the movements, like motor neurons or cortical neurons in the motor cortex) was even intuitively easy to imagine because each active neuron can be represented as a vector, with Direction (as its preferred movement direction) and Magnitude (as its firing rate). Higher firing contributes more to the final movement direction. This concept was introduced by Georgopoulos et al. (1981, 1982, 1986), and at first, it was a way of describing how groups of neurons in the motor cortex encode the direction of limb movements.

## 4 Spatial coding of 2D-arm movement direction by neuronal populations in the primate cortex

**FIGURE 3:**
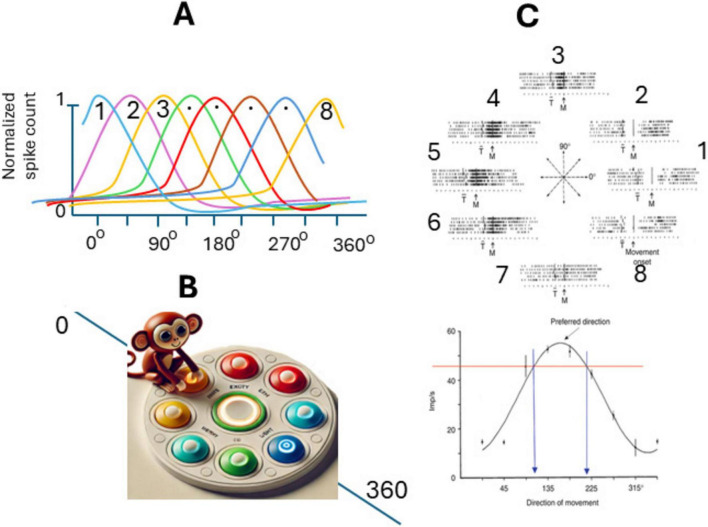
Spatial coding of movement direction by populations of neurons in primary motor cortex (M1). **(A)** Tuning curves of eight random neurons in M1 with preferred directions spanning 360 degrees. **(B)** Experiment setup, the animal (monkey) is positioned in front of eight-LED located on a circle with an 8 cm radius, and one additional LED in the center. The monkey is trained to move a special manipulandum to touch the lit button. **(C)** Orderly variation in the frequency of discharge of a motor cortical cell with the direction of movement: Upper panel impulse activity in the population of neurons in M1 during five repetitions of movements made in each of the eight directions indicated by light: the eight directions indicated by the center diagram. Notice the orderly variation in the cell’s activity. Lower panel Directional tuning curve of the same cell [from Georgopoulos et al. (1982) with permission].

In the initial experiments of the authors (Georgopoulos et al., 1981, 1982), a rhesus monkey was positioned in front of a table with 8-LED (light emitting diodes) positioned on a circle; and one additional LED in the center (Figure 3B) each can be lit by the experimenter. Also, a special manipulandum allowed the monkey to move it in 2D and point with it to the activated light (capture it). The animal was trained first to capture the center light and held that position for a few s; then a peripheral LED came on and the animal had to capture it with the freely movable manipulandum moved by the monkey’s arm to receive a rewards. The activity of single cells in the primary motor cortex (M1) was recorded while monkeys made arm movements in eight directions in this two-dimensional apparatus, each starting from the same point. The frequency of discharge of about 75% of cells from the M1 active during movements (during the reaction time, the movement time, and the period that preceded the earliest changes in the electromyographic activity approximately 80 ms before movement onset), varied in an orderly fashion with the direction of movement (Figure 3C). Discharge was most intense with movements in a preferred direction and was reduced gradually when movements were made in directions farther and farther away from the preferred one. This resulted in a bell-shaped directional tuning curve. The authors decided that it could be significantly fit by a cosine function for each neuron. Preferred directions differed for different cells so that the tuning curves partially overlapped (Figure 3A). Interestingly, some neurons were active not only during, but also before movement onset, about 64% of the cells were activated before the earliest electromyogram changes in muscles and 87% before the onset of a movement in the cells’ preferred direction, predicting future movements. Also, these neurons responded to passive movements of the animal arm by experimenters, but responses to passive manipulations were less pronounced and complex–were evoked conditionally, and depressed after a few stimuli (Georgopoulos et al., 1982).

Thus, Georgopoulos et al. (1982) recorded motor cortex neurons in rhesus monkeys (M1) performing two-dimensional reaching movement and found that many neurons were active for multiple movement directions but showed maximal firing for specific directions (Georgopoulos, 1994). Also, by computing the weighted sum of neuronal responses, they could predict the overall movement direction with high accuracy. The formula used to calculate the overall direction was the same vectorial summation as the one for the sensory neuron populations, while the number of participating cells was much larger (Georgopoulos et al., 1986). It became clear that other neurons in the brain that controlled directional movements were similarly active for multiple movement directions but showed maximal firing for specific directions. In earlier works, for simplicity, this vector function was assumed to be cosine-shaped; however, it was later clarified that such a function is much more compact and more accurately corresponds to a circular Gaussian (the so-called von Mises distribution) with a single peak, although in 16% of cases it is bimodal, meaning it has two peaks (Amirikian and Georgopulos, 2000).

Besides M1, many neurons in different parts of the brain controlling movements also possessed preferred directions. It was found that neurons in the superior colliculus (SC) which displayed saccade-related spike activity, have movement fields, with each cell discharging in association with rapid eye movements that have a particular range of directions and amplitudes (Lee et al., 1988), and it shares mechanisms with glissades (Chen et al., 2015). Vector-average spatial representation was demonstrated by readout of the rostral SC controlling microsaccade execution (Hafed and Ignashchenkova, 2013). Similarly, population coding of movements was found in the premotor cortex (Panzeri et al., 2015). The population vector model demonstrates that the preferred direction is an emergent property of the collective activity of neurons. It is widely used in brain-machine interfaces (BMIs) for decoding intended movements from neural activity (Wessberg et al., 2000; Carmena et al., 2003).

It was also found that M1 firing in addition to the well-studied average directional selectivity (“preferred direction”) of single-cell activity, was also correlated with the time-varying speed of movement and is encoded in the same neurons controlling the directional information (Moran and Schwartz, 1999a). Also, populational M1 cortical activity was found to correlate with arm position in three-dimensional space (Kettner et al., 1988), acceleration (Flament and Hore, 1988), target position (Alexander and Crutcher, 1990; Georgopoulos et al., 1989) and joint configuration (Scott and Kalaska, 1997). Each neuron participated in different activities simultaneously. If the direction of movement was intuitively simple to be interpreted as vector direction, other features would require a more “mathematical” interpretation of vector, focusing on “preferred input.” The formula for calculating the (normalized) population vector F takes the following form:


$$F=\frac{\sum_{i}m_{i}\times F_{i}}{\sum_{i}m_{i}}$$

Where *m_i_* is the activity of cell *i* and *F_i_* is the preferred input for cell *i*j. It can be “preferred” direction, preferred speed of movement, preferred joint angle, etc.,

Interestingly, this broad understanding of neuronal preferences brings a wider understanding of the sensory and motor fields of individual neurons and the influence of the number of neurons in the coding population. In both visual and motor cortex models, the population vector leverages the bell-shaped tuning curves of individual neurons to encode a variable. In theory, this variable can be fully recovered using only a small number of neurons. Without response variability, just a few neurons are sufficient to represent the entire range of orientations, thus some part of the population can be removed (for example, by applying a local anesthetic). This property resembles the effect seen in holography, then the image may be restored in full by a small part of the hologram (Sokolov and Vatkyavichus, 1988). The data supports the distributed coding hypothesis, where motor control emerges from population dynamics rather than single neurons.

Interestingly, it was found that the distribution of preferred directions in M1 is correlated to mechanical anisotropies of the limb and may be more complex (Scott et al., 2001). Some authors even tried to explain the emergency of population vectors as a result of different whole-limb motor tasks, under the assumption that cortical neurons encode low-level muscle activation and that the conversion of muscle force to hand motion depends on the geometry of the limb, its inertial properties and the presence of external loads, suggesting Jacobian linear model (Todorov, 2000). This model assumes that each pyramidal tract neuron contributes additively, either via direct projections onto motor neurons or indirectly through spinal interneurons, to the activation of muscle groups (Todorov, 2000; Scott, 2000). While joint biomechanics introduce non-linearities incompatible with the simple Jacobian assumption (Scott, 2000), it is clear that most proposed limb movement parameters are interconnected through fundamental physical laws. For example, in the limb, if all segments (bones) are connected with joints, the movement of all parts is interdependent.

## 5 The movement vector of the limb interconnected by joints is a vector sum of the movements of its components

**FIGURE 4:**
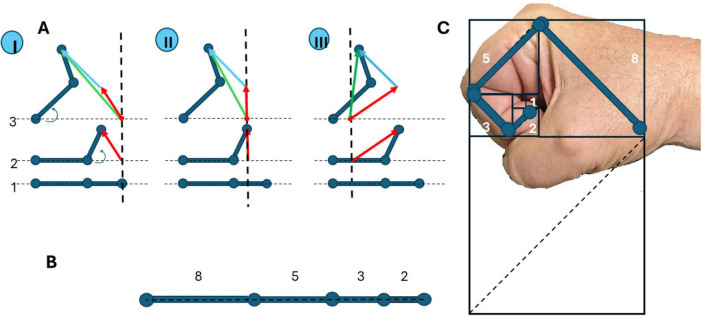
Movements of the limb and its components interconnected by joints. **(A)** Movement of the limb that consists of two bones interconnected by the joint, presented in three coordinate origins (I, II, and III): 1-all bones are in line, two-movement of the first bone (marked as a red arrow), 3-movement of the second bone (marked as a green arrow). Summary movement is always the sum of movements of the first and second bones (marked as a blue arrow). **(B)** The arm has bones of different lengths, related to Fibonacci numbers 2-3-5-8 [approaches the Golden Ratio (approximately 1.618)] as the sequence progresses. **(C)** Fibonacci numbers as the length of bones in a human hand.

Suppose the limb consists of two segments (bones), interconnected by the joint, like in a simple arm (Figure 4A). Let initially these two bones be in one line (Figure 4AI). Then one bone is moved (Figure 4AII), this bone movement can be marked as a vector (red arrow). Then another bone is moved (Figure 4AIII), this bone move can be marked also as a vector (blue arrow), and the sum of bone movements is, actually, the whole limb movement and can be marked as a summary vector (green arrow). These movements are present in three different coordinate origins, showing that the vector representing two bone movements (which are components of the whole movement) is always the sum of component movements, and does not depend on coordinate origins. Similarly, it does not depend on the number of components united by joints, there may be any quantity of joints (Figure 4B).

Interestingly, the human upper limb, comprising sequentially connected bones–humerus, radius/ulna, and metacarpals–functions as a biologically optimized manipulator. Notably, the relative lengths of these segments often approximate a Fibonacci sequence, wherein each number is the sum of the two preceding terms (0, 1, 1, 2, 3, 5, 8, …) (Figure 4B). This mathematical structure, beyond its esthetic and natural appeal, underlies powerful optimization principles, which may explain its recurrence in biological systems. In algorithmic theory, Fibonacci numbers are known for their role in efficient solutions to the coin change problem. According to Zeckendorf’s theorem (Pooksombat et al., 2022), any positive integer can be uniquely represented as the sum of non-consecutive Fibonacci numbers. This property enables a “greedy algorithm” to construct such sums optimally by iteratively selecting the largest possible Fibonacci number that does not exceed the remaining value. In practical terms, this ensures minimal component usage–an efficient solution mirrored in certain national coinage systems and computational strategies. Analogously, a jointed manipulator–biological or robotic–may achieve movement efficiency if its segments follow Fibonacci proportions. In such a system, reaching a target in space can be viewed as a decomposition task: the end-effector’s position is composed of vector segments contributed by individual joints. A Fibonacci-based length distribution enables the use of a greedy motion algorithm, prioritizing longer segments first and progressively incorporating shorter ones. This strategy minimizes the number of joints actively engaged in a movement, conserving both neural control effort and mechanical energy (Figure 4C). Thus, the Fibonacci sequence may confer biomechanical advantages in natural manipulators such as the human arm. The Fibonacci-like scaling of ossial lengths furnishes a geometric backbone that proportionally balances leverage across successive joints, allowing population-vector motor commands to map more linearly onto limb torques and thereby simplifying neural control of the entire extremity. By enabling economical and versatile movement through a minimal set of joint activations, it likely represents an evolutionary convergence of form and functional efficiency. Also, the cohesion of manipulator elements produces interdependence, and the overall movement is always described by individual vector summation.

The population vector analysis was used in the study to extract information encoded in a population of motor cortical neurons recorded during the performance of individual fingers and wrist movements, a manipulator with advanced kinematic structure (Georgopoulos et al., 1999). Movements were examined using data from Schieber and Hibbard (1993), which demonstrated that cortical neurons do not respond selectively to the flexion or extension of individual fingers but rather become active during movements involving all fingers or their specific subsets. Moreover, the neuronal populations engaged in several finger group movements exhibit overlapping spatial distributions within the motor cortex. The discharge of motor cortex neurons related to distinct finger movements contains information representing the spatial geometry of the hand embedded within the flexion and extension movement domain. The authors applied population vector analysis and found that a majority of individual neurons (132 out of 176) were “tuned” to specific movement directions within the space of wrist and finger motion. Furthermore, the computed population vector closely matched the actual direction of finger movement. Similar findings were reported by Moran and Schwartz (1999b), who recorded from individual neurons in the premotor cortex of monkeys. In this study, the animal used its index finger to trace various shapes (e.g., circles, squares) displayed on a touch-sensitive screen. Neural activity was recorded continuously and analyzed as a single population, drawing from four cortical areas (three primary motor regions and one dorsal premotor area). The resulting population vector accurately reproduced the shapes being traced. Temporal relationships between neural activity and finger movements were also assessed, revealing that neural responses did not consistently precede movement onset by a fixed delay. The movements consisted of four kinematic segments clearly distinguishable in the population vector analysis. The population vector successfully predicted both the direction and velocity of finger motion. In another study, (Caminiti et al., 1991) investigated the activity of 156 individual neurons in area 6 of the monkey premotor cortex during arm movements performed within a three-dimensional sphere. The vast majority of neurons (152, or 97%) showed systematic modulation of their activity across different movement directions, exhibiting clear directional preferences. These preferred directions formed a continuous representation across 3D space. However, when the animal was required to move its hand to the same final position from different starting locations, individual neurons adjusted their directional tuning vectors. These shifts varied in magnitude across neurons and were particularly evident during elbow rotations, suggesting that animals selected the most efficient trajectory to reach the target. Similar tuning vector changes were also observed in the primary motor cortex. In contrast to individual tuning vectors, the population vector remained stable and did not shift with joint rotations. Neuronal activity in both motor and premotor cortices was significantly influenced by the static starting position of the arm. Nevertheless, in both motor and premotor areas, the overall population vector reliably predicted the direction of overall movement.

While a neuronal population vector with “preferred input” may be enough to describe many sensory and motor-related neuronal vectors, a more profound model would have additional advantages.

## 6 Motor neurons as vectors

In physics and engineering, vectors are commonly used to represent quantities that depend on multiple variables. Formally, a vector is an array or list of numbers, known as components, which describe its behavior across different dimensions or variables. The activation of motoneurons can be similarly represented using vector principles, particularly in how signals from neurons combine to produce a resultant effect. Each motoneuron can be conceptualized as a vector, where the magnitude represents the level of motor activation or the strength of the signal transmitted through its synapses. Since a motoneuron can form different connections, its magnitude can vary across synaptic contacts, making it multidimensional. The direction of the vector corresponds to the specific muscle or motor field that the neuron innervates, which can also span multiple dimensions. When two motoneurons partially overlap in their motor fields, their effects can be visualized as the addition of vectors. If one motoneuron is strongly activated while the other is moderately active, the combined effect is represented by the resultant vector, which is the sum of their individual contributions. This process mirrors vector addition in physics, where the components of the vectors combine to produce a resultant vector. The point at which a muscle reaches its contraction threshold corresponds to the resultant vector’s magnitude exceeding a specific threshold. Just as in physics, where a resultant force vector must surpass a certain magnitude to produce a physical effect, in neurophysiology, the combined activation from two or more motoneurons must exceed a threshold to trigger muscle contraction (Theeuwen et al., 1994). These established findings allow for the conversion of activity level to direction because muscles themselves have anatomical preferred directions (Schieber and Hibbard, 1993).

## 7 Motor units in the mammalian muscle have a broad range of tuning, a unit direction changes gradually depending on its location within the muscle

While anatomical preference direction in muscle contraction seems obvious, it happens that the directional activity of muscles is broadly and often multimodally tuned: one muscle as a whole has a fairly broad tuning range for the direction of the force it produces. Herrmann and Flanders (1998), (Schieber and Hibbard, 1993) measured the “best” force directions of motor units in the biceps (both heads) and the deltoid muscle (anterior, middle, and posterior parts) in humans by recording individual motor units during isometric exertion in various directions (Herrmann and Flanders, 1998). For all muscles studied, neighboring motor units could have significantly different best directions, suggesting that each muscle receives multiple directional commands (Herrmann and Flanders, 1998). However, it was found that each motor unit has its own best direction, which does not coincide with the best directions of other motor units within the same muscle, and that 17% of motor units have two or more best directions. The best direction of a motor unit changes gradually depending on its location within the muscle. The directional vectors did not cluster into any groups. It is suggested that central mechanisms, when recruiting motor units in a muscle, also take into account the “best” directions specific to each unit. Thus, the direction of muscle movement depends on the population of motor units participating in the movement, and the population of motor neurons controlling these units. We may predict that summary movement will be the vector summation of the population.

## 8 The combined effect of motoneurons in snails

Snails, particularly those in the genus Helix, have been significant in neurophysiological research due to their relatively simple nervous system compared to higher animals, making them easier to study and understand, and having large, identifiable neurons accessible for experimental manipulation. These neurons, such as the giant motor neuron C3 in the cerebral ganglion, or LPa3 in the pleural ganglion of Helix, are large enough to be easily recorded from and stimulated, providing valuable insights into the neural function, motor effect, and behavior (Munoz et al., 1983; Iniushin et al., 1987; Bugai et al., 2005). Snails also exhibit a range of relatively complex behaviors, including feeding, mating, and withdrawal reflexes, which can be studied in detail by analyzing neural circuits.

**FIGURE 5:**
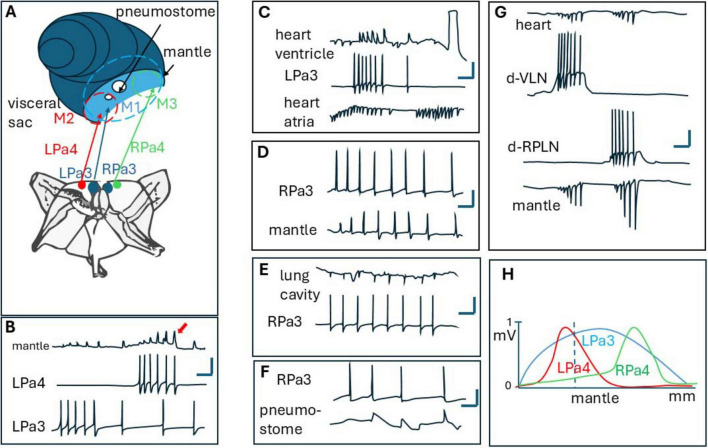
**(A)** position of neurons LPa3, Lpa4, Rpa3, Rpa4 in the right and left parietal ganglion of Helix. Also, motor fields of LPa3 (M1), Lpa4 (M2), and RPa4 (M3) are shown (as dashed circles) near the mantle and pneumostome. Note, that all these motor fields are superimposed. **(B)** Synaptic potentials recorded from the mantle, in superimposed part of LPa3 and LPa4 motor fields. Note summation of SPs (arrow), **(C–F)** synaptic potentials elicited by LPa1 or RPa3 neurons in the mantle, lung cavity, heart, and around pneumostome in Helix [from Iniushin et al. (1987), with changes]. **(G)** Synaptic potentials in mantle and heat from giant neurons d-VLN and d-RPLN in Achatina [from Safonova et al. (2008)], **(H)** distribution of motor fields (studied as normalized synaptic potentials amplitudes along Helix mantle near pnemostome). Note, that LPA3 controls all the mantle as a whole, while LPa4 controls the right, and RPa4 controls the left part of it.

Snails have no internal skeleton, and muscles in these animals have no specific reciprocal control, like in animals with skeletons (vertebrates and arthropods), however, they possess a sophisticated hydraulic system that participates in tentacle and body movements (hydraulic skeleton). The absence of reciprocal control makes the analysis of snail movements easier and can be described as a summation of the effects of participating motoneurons on their motor fields. On the other hand, snails possess motoneurons with large motor fields that span both myocardium control (providing hydraulic pressure) and body wall, pneumostome, and tentacle muscles (Bugai et al., 2005; Safonova et al., 1984; Zhuravlev et al., 1989) (see Figures 5A–H).

The motoneurons and their activation in snails can be described using vector principles. Each motoneuron can be thought of as a vector, where the magnitude of the vector represents the level of activation or the strength of the signal sent by the motoneuron, and the direction represents the specific motor field (or multiple motor fields) it innervates, thus determining the particular direction (or various directions) of muscle movement. When two motoneurons have partially superposed motor fields, their effects can be considered as vector sum. This is similar to how vectors are added in physics, where the resultant vector is the sum of the components of the individual vectors (Taylor, 2025). The muscles reaching the contraction threshold can be seen as the point where the resultant vector (the combined activation from both motoneurons) exceeds a certain magnitude.

For example, giant cholinergic motoneurons LPa3 and RPa3 (from left and right parietal ganglions in Helix) control muscles from the lung cavity (Figure 5E), mantle (Figure 5D), pneumostome (Figure 5F), and heart (Figure 5C). Interestingly, these neurons activate only the ventricle, not the atrium in the heart (Figure 5C), thus controlling the hydraulic skeleton as well. LPa3 and RPa3 motor fields overlap significantly. On the other hand, smaller motoneuron LPa4 (its cell body is situated near LPa3, and marked red in Figure 5A) has a smaller motor field, which also overlaps with the motor field of LPa3. On the mantle and body wall near the pneumostome one can see that the motor field M1 of LPa3 (blue) is overlapped with motor field M2 (red) of LPa4. Muscles in the overlapping regions generate postsynaptic potentials (PSPs) in the muscles (Figure 5B) which can be recorded using flexible plastic suction electrodes (Iniushin et al., 1987). A comparison of postsynaptic potentials from different neurons at the same point showed that postsynaptic potentials from neuron RPa4 appeared in the mantle muscles 25–30 ms earlier than those from RPa3 (Zhuravlev et al., 2004). Activation of the muscle reaching the contraction threshold can be achieved by high-frequency stimulation from each of the motoneurons, or by the addition of stimulus from both motoneurons, working simultaneously (Figure 5B, arrow). Interestingly, because high-order motoneurons in snails participate in a variety of movements, and their hi-frequency discharge leads to “escape” contraction of main muscles leading to the hiding of the animal inside its shell, these cells were deemed “command” neurons and even the concept of command neuron arises (Balaban and Litvinov, 1977; Kupfermann and Weiss, 1978). Besides LPa3 and RPa3 in *Helix pomatia*, similar motoneurons play crucial roles in coordinating specific behaviors in other snails. For instance, in the tentacle withdrawal reflex of *Helix aspersa*, the giant motor neuron C3 is paramount in eliciting and forming both tentacle retraction and bending (Prescott et al., 1997).

In the whole-body withdrawal reaction of snails, biphasic excitation of motoneurons can arise from sensory stimulation or spontaneously, indicating a central program that can be triggered and modulated by feedback from motoneurons (Arshavskii and Deliagina, 1990). Of course, hi-order motoneurons can themselves be interneurons in another reflex arc: In Helix, the tentacle withdrawal reflex involves the activation of motor neurons in the tentacle ganglion, which then send signals to the cerebral ganglion to activate the giant motor neuron C3 and other smaller motor neurons. This coordinated action results in both tentacle retraction and bending (Hernádi et al., 2014). In the posterior tentacles of snails, local motor neurons can be activated by peripheral stimuli, such as olfactory inputs, to generate local movements without the direct involvement of the central nervous ganglions (CNS). These local motor neurons can receive inputs from local interneurons, modulating their activity to produce patterned contractions (Hernádi et al., 2014). In swimming mollusks neurons controlling cardiac functions also overlap with locomotor neurons in certain cases. For example, heart excitatory neurons (HE) and wing motoneurons exhibit coordinated activity during increased locomotion (Kodirov, 2011). While not explicitly described as vector summation, this coordination reflects how overlapping motor fields can produce integrated physiological responses.

Of course, in addition to the simple summation of the motor field of motoneurons, snails coordinate muscle contractions through a complex interplay of central and peripheral mechanisms, involving specific neural circuits and feedback loops. For repetitive behaviors, like feeding and locomotion, an important central component called central pattern generator (CPG) was found which may generate rhythmic patterns that directly control motoneurons. For example, during feeding in snails like Helisoma, the buccal ganglion contains CPGs that activate distinct groups of motor neurons during different phases of the feeding cycle (protraction, rasp, and swallow) (Murphy, 2001; Barkan and Zornik, 2019). These motor neurons are often electrically coupled to interneurons, ensuring synchronized activity (Barkan and Zornik, 2019; Staras et al., 1998).

Also, snails move by generating rhythmic muscular contractions, known as pedal waves, on the underside of their foot. These waves push the snail’s body forward by creating friction between the foot and the surface. The foot secretes mucus, reducing friction and allowing the snail to glide smoothly over various surfaces. The waves alternate between muscular contractions and relaxations, creating a forward motion as different parts of the foot push against the substratum. In sea mollusks rolling pedal waves produce undulations of the body allowing axial locomotion. The generation of pedal waves in mollusks, such as Aplysia, involves a single central pattern generator (CPG). Research on Aplysia locomotion reveals that rhythmic pedal waves are produced by a coordinating activity through two phases (phase I and II) mediated by interneurons and electrically coupled motoneurons. The motor neurons (P1Ns) involved in locomotion exhibit phase-specific activity during two distinct phases of the motor program: Phase I (168°) involves class 1 interneurons (PI1/PI2) driving ipsilateral motoneurons via strong electrical coupling, while Phase II (357°) involves class 2 interneurons (PI3) synchronizing contralateral activity. Computational analyses suggest that the pedal ganglion may function as a spiral attractor network, which integrates motoneuron activity to generate smooth, propagating pedal waves. This could be interpreted as a vector summation of motoneuron signals across overlapping motor fields (Chen et al., 2012; Wang et al., 2023). In Clione mollusks, there are two special (A1 and A2 from pedal ganglion) neurons responsible for generating a frequency of pedal waves. Neurons 1A and 2A fired reciprocally at the beginning of the phase of elevating and lowering the wing, respectively (Arshavsky et al., 1985). Similarly, it may be interpreted as a vector summation of two frequencies.

Although mollusk studies do not explicitly describe motoneurons as vectors in the mathematical sense, their functional organization–such as overlapping motor fields, electrical coupling, and phase-specific activation–aligns well with the concept. The integration of motoneuron signals within CPGs and neural circuits produces coordinated movements akin to vector summation in physics.

## 9 Discussion of recent works and future projections

While this review concentrated on the foundations of vectorial ideas in neurobiology, our survey shows that population-vector coding is more than a convenient read-out; it is deeply entwined with the brain’s predictive-coding machinery. In predictive coding, the brain continually compares incoming signals with internal forecasts, passing forward only the mismatch (prediction error). Recent high-density Neuropixels and two-photon studies (Failor et al., 2025; van Beest et al., 2024; Paulk et al., 2022) reveal that population vectors rotate through low-dimensional manifolds that anticipate the animal’s next sensory state or motor outcome–exactly the behavior expected if vectors embody predictions that are updated on the fly.

This predictive role has direct consequences for brain–machine interface (BMI) design, which uses vectoral representation of movements in the brain as a predictive “model” that needs to be extracted. Besides the sensitivity to instabilities at the neural interface resulting in a degradation of decoding performance, decoders that treat population vectors as static “output channels” ignore the anticipatory drift embedded in neural state space; by modeling that drift, future adaptive BMIs can reduce latency and improve accuracy. Non-linear Manifold Alignment with Dynamics (NoMAD), uses unsupervised distribution alignment to update the mapping of non-stationary neural data to a consistent set of neural dynamics (Karpowicz et al., 2025)

The biomechanical regularities highlighted by Verrelli et al. (2021)–Fibonacci-like scaling that linearizes limb torque mapping–further imply that optimal prosthetic actuation should respect native geometric ratios, ensuring that decoded neural vectors translate into naturalistic forces. Finally, emerging links between population-vector dynamics, self-organized criticality, and fast oscillatory synchrony raise the possibility that critical-state network models may become valuable priors for both decoding algorithms and artificial neural reservoirs (Bak et al., 1988; Bai et al., 2016; Yu et al., 2008; Nguyen et al., 2021).

Future extensions of this review should therefore cover:

• Multichannel and optical recordings that expose predictive vector trajectories in real time.
• Single- versus multi-site microstimulation protocols for writing vector “priors” back into the cortex.
• Parallels between one-bit LLM updates and spike-based weight adjustments in biological vectors.
• Roles of self-organized criticality and mesoscale synchrony in stabilizing long-range vector coherence.

## 10 Conclusion and outlook

Population-vector coding provides a predictive, low-dimensional language that the nervous system re-uses whenever high-dimensional activity must be transformed into behaviourally relevant commands. Modern recording and stimulation technologies reveal that these vectors are dynamic forecasts, tightly coupled to body geometry and nested within oscillatory and critical-state network architectures. Open questions include: How are vector predictions combined across cortical levels? What network motifs maintain criticality without sacrificing stability? How many artificially injected “votes” are required to bias a native population vector? And can one-bit or reservoir-computing hardware exploit the same principles to achieve energy-efficient inference?

Translational opportunities span closed-loop prosthetics that predict user intent before movement onset, optogenetic or electrical feedback that embeds sensory priors directly into cortex, and neuromorphic chips that mimic vector-based predictive coding for edge AI. Bridging these basic and applied avenues promises both deeper insight into neural computation and practical gains for neuro-rehabilitation and human–machine symbiosis.
