# Supplementary material for: Vectorial principles of sensorimotor decoding
Source: Front Hum Neurosci. 2025 Jul 7;19:1612626. doi: 10.3389/fnhum.2025.1612626 (PMC12287768; doi:10.3389/fnhum.2025.1612626)
Supplement: Supplementary file 1 [file Table_1.docx]

# Supplementary Table S1. Glossary

| Term | Brief explanation |
| --- | --- |
| Mayer and Young’s Color Triangle | 19th‑century triangular diagram showing colour mixtures of three primaries; basis for trichromacy. |
| Automorphisms | Transformations that map a mathematical object onto itself while preserving its structure. |
| Opsins | Light‑sensitive retinal proteins that initiate phototransduction when they absorb photons. |
| Microspectrophotometry | Method for measuring absorbance spectra of tiny samples such as single photoreceptor cells. |
| Dichromatic | Possessing two cone classes and therefore two spectral channels for colour vision. |
| Early sensory neurons | Primary receptor neurons that transduce external stimuli before central synaptic processing. |
